# Exploring experiences of mental health challenges in under-represented young people (aged 16–24 years) in England: a narrative inquiry protocol

**DOI:** 10.1136/bmjopen-2024-098223

**Published:** 2025-11-26

**Authors:** Rebecca Syed Sheriff, Jason Arday, Rohit Shankar, Roisin Mooney, Louise Chandler, Helen Adams, Lili Z Nagy, Roger Farrell, Daisy Fancourt, Scott Weich, Catherine Henderson, Shaima Hassan, Joe Langley, Kamaldeep Bhui

**Affiliations:** 1 University of Birmingham, Birmingham, UK; 2 Department of Psychiatry, University of Oxford, Oxford, UK; 3 Faculty of Education, Cambridge University, Cambridge, UK; 4 Plymouth University Peninsula Medical School, Truro, UK; 5 CIDER, Cornwall Partnership NHS Foundation Trust, Truro, UK; 6 University of Oxford, Oxford, UK; 7 University of Plymouth, Plymouth, UK; 8 Department of Behavioural Science and Health, Department of Epidemiology and Public Health, University College London Research, London, UK; 9 School of Health and Related Research, University of Sheffield, Sheffield, UK; 10 London School of Economics, London, UK; 11 University of Liverpool, Liverpool, UK; 12 Lab4Living, Sheffield Hallam University, Sheffield, UK

**Keywords:** Health, Adolescents, MENTAL HEALTH

## Abstract

**Abstract:**

**Introduction:**

Three-quarters of mental health problems start before the age of 25. However, young people are the least likely to receive mental healthcare. Some young people (such as those from ethnic minorities) are even less likely to receive mental healthcare than others. Long-term impacts of mental health problems include poorer physical health, relationships, education and employment. We aim to elicit the views, experiences and needs of diverse young people (aged 16–24 years), to better understand (1) their experiences of under-representation, mental health and coping, (2) mechanisms that shape mental health trajectories and (3) how online arts and culture might be made engaging and useful for young people’s mental health. We also aim to do this with autistic young people.

**Methods and analysis:**

Narrative inquiry will be employed as a tool for gathering young people’s perspectives for an iterative analysis. The narrative method proposes that critical insights and knowledge are distributed across social systems and can be discovered in personal stories and that knowledge can be relayed, stored and retrieved through these stories. Data will be transcribed and explored using a combination of thematic and intersectional analysis. Young people will be core members of the research team, shape the research and be involved in the coding of data and interpretation of the findings.

**Ethics and dissemination:**

This study (IRAS project ID 340259) has received ethical approval from the HRA and Health and Care Research Wales (REC reference 24/SC/0083). The outputs will identify touch points and refine the logic model of how online arts and culture might support the mental health of those from under-represented backgrounds. We will share knowledge with young people, policy makers, health professionals, carers, teachers, social workers and people who work in arts and culture. We will produce research papers, blogs, newsletters, webinars, videos and podcasts.

**STRENGTHS AND LIMITATIONS OF THIS STUDY**

- Narrative inquiry will enable under-represented young people to tell their stories in their own way leading to increased insights into their experience of mental health challenges.
- An intersectional lens will be used to understand the perceived needs of young people of diverse identities and diverse place contexts, allowing for a deeper and more nuanced understanding of those facing multiple disadvantages.
- The extent of lived experience involvement throughout (as advisors, peer researchers, coordinators, participants and investigators) will contribute to ensuring youth-informed research.
- Challenges will include engaging young people of diverse identities, who are usually under-represented in research, in sharing authentic narratives.

## Introduction

 Three-quarters of mental disorders emerge before the age of 25,^1 2^ yet young people aged 16–24 years are the least likely age group to receive help from mental health services.^3 4^ Common mental health problems in young people, such as depression and anxiety, have long-term sequelae: on mental and physical health, social support, relationships, employment, as well as health risk behaviours including self-harm.^5 6^ There are significant direct and indirect costs over the life course.^1^ The COVID-19 pandemic and associated restrictions disproportionately impacted on the lives of young people.^7^ Following the pandemic, demand for mental healthcare has increased and access diminished.^8 9^ Some young people already face greater challenges and are affected more than others, for example, those identifying as ethnic minority and/or Lesbian, Gay, Bisexual, Transgender, Questioning and other sexual orientation or gender identities.^4 10 11^ Many autistic young people lack suitable psychosocial support, compounding mental health inequalities, yet there is little research into their mental health needs or how best to engage them in research as equal partners.^12 13^ Thus, there is a compelling need for evidence-based, accessible and engaging resources to support the mental health of young people.^14^ This is a priority for young people, health services, social care, education, employment and societal recovery.^15^,^17^

We consulted people with lived experience of mental health challenges regarding the potential benefits of online arts and culture. Online arts and culture was defined as online content provided by museums, theatres, art galleries, libraries, archives and natural heritage organisations. Young people expressed that online arts and culture could be effective in improving mental health and reducing symptoms of depression and anxiety, that it could be accessed regularly, remotely and as needed to aid self-management and provided a positive alternative to social media.^18^

We undertook a literature review,^19^ conducted an online survey into the use and potential benefits of online culture and the arts^20^ and investigated the potential mechanisms of mental health benefits of online arts and culture through qualitative studies and codesign with young people aged 16–24.^18^ In particular, human stories were identified as the single most important ingredient for mental health benefit. Participants recommended more content describing the experiences of a range of people from different backgrounds that they could connect with on a human level (reinforcing positive identities/bonding social capital),^21^ and more alternative viewpoints (shifting thoughts, promoting curiosity and learning; bridging social capital).^22^

We then coproduced an online intervention for mental health in young people*,* based on the diverse human stories of the people behind art and artefacts called Ways of Being (WoB). In a randomised controlled trial^23^ comparing WoB with a typical museum website (the Ashmolean Website, Ash), we recruited 463 people aged 16–24. Most of them were recruited within 2 weeks of the study first being advertised; 74% of participants provided outcome measures to 6-week follow-up. At baseline, 89% of participants self-reported levels of psychological distress consistent with clinically significant depression and/or anxiety. Psychological distress and negative affect reduced over the intervention phase compared with baseline in both groups. Negative affect was lower in those allocated to WoB than those allocated to Ash (n=448, –0.158, p=0·010). However, WoB group participants said that they ran out of content to look at and group differences were not sustained at 6 weeks. Group differences were greater for participants of minority ethnicity. 90% of participants said they would be keen to take part in future research. A short video of our preparatory work is available online.^24^

The idea that culture and the arts are good for mental and physical health and well-being is not new.^25^,^28^ The mechanisms that improve mental health include emotional activation, aesthetic engagement, interaction, social and cognitive stimulation, sensory activation and imagination.^28 29^ However, engagement with culture and the arts is poorest for those who might benefit the most.^20^ During consultations in our work, people with lived experience of mental illness said that this was not due to lack of interest.^30^ They thought that those with lived experience should be an active part of any strategy to optimise such resources for mental health benefit. Building on this, we have worked with stakeholders and young people to develop the study described in this protocol. A walkthrough video is available for WoB.^31^

The following research questions apply to young people aged 16–24 in work package 1 (WP1) and to autistic young people in WP2, aged 16–24 years, living in England.

- What are young people’s experiences of mental health, resilience, recovery and formal and informal care?
- What are the mechanisms that involve identity, place and health beliefs that shape distinct trajectories of mental health?
- How might online mental health interventions based on arts and culture be engaging and made useful for young people at the intersections of multiple disadvantages (through identity, place, deprivation, mental health, etc)? What adjustments are needed for autistic young people?

## Methods and analysis

This research informs part of the discovery phase of a larger programme of research funded by the NIHR called ORIGIN (Optimising Cultural Experiences for Mental Health in Marginalised Young People Online). This research addresses 5 of the top 10 mental health research priorities identified by young people.^32^ In accordance with the Medical Research Council principles of developing complex interventions,^33^ we aim to continue to identify and understand mechanisms of online arts and culture on young people’s mental health and symptoms of depression and anxiety in even more diverse populations.^30^

To gather detailed information, we use a narrative inquiry tradition and an intersectionality lens to address the needs of young people of diverse identities and from diverse place contexts, facing multiple disadvantages (see table 1). The narrative inquiry tradition will be employed as a tool for gathering young people’s perspectives,^34^ and for iterative analysis in a carefully designed study. The narrative method proposes that critical insights and knowledge are distributed across social systems and can be discovered in personal stories. The knowledge can be relayed, stored and retrieved through these stories.

**Table 1:** Research design of each study

	Work package 1 (WP1)	WP2
Design	We will use qualitative methodologies to gain detailed experience and mechanistic information to inform codesign in a later study of a large research programme. To gather this detailed information, we will use a narrative inquiry tradition and an intersectionality lens to understand the perceived needs of young people of diverse identities and diverse place contexts, facing multiple disadvantages.	We will use qualitative methodologies to gain detailed experience and mechanistic information to inform codesign. To gather this detailed information, we will use a narrative inquiry tradition and an intersectionality lens to understand the perceived needs of autistic young people with enhanced needs, facing multiple disadvantages. Particular focus of WP2 is on reasonable adjustments needed for the future studies to be inclusive of autistic young people.
Setting	Community, NHS waitlist and online: We will recruit from communities (rich in under-represented young people) and NHS waitlists focussing on Sheffield, Oxfordshire, Cornwall/Devon, Blackpool and Liverpool and online from any region of England. We will select a minimum of 100 participants via purposeful sampling (see box below).	We will recruit via the University of Plymouth’s Cornwall Intellectual Disability Equitable Research centre and eligible young people recruited via WP1 who have consented to further contact and meet the eligibility criteria but are unable to be selected for WP1 as they have enhanced autistic needs (level 2 autism).
Sample	We will select (n=100) young people aged between 16 and 24 years with current or previous mental health difficulties via purposeful sampling based on under-representation (by ethnicity, sexual identity and orientation, neurodiversity, geography, occupation, education and deprivation), use of online arts and culture and previous help-seeking	We will select autistic young people aged between 16 and 24 years with current or previous mental health difficulties via purposeful sampling on the basis of under-representation (by ethnicity, sexual identity and orientation, occupation, education and deprivation), use of online arts and culture and previous help-seeking
Inclusion criteria	Participant is willing and able to give informed consent for participation in the study. Male, female or non-binary, Aged 16–24 years Identifies as having current or previous mental health difficulties in the past year OR scores ≥20 on the K10 Identifies as feeling under-represented in some way, for example, by ethnicity (eg, ethnic minority), sexual identity or orientation (LGBTQIA+), neurodiversity (eg, ADHD), occupation or education (eg, not in education, employment or training) or deprivation	Participant is willing and able to give informed consent for participation in the study. Male, female or non-binary Aged 16–24 years. Autistic Identifies as having current or previous mental health difficulties OR scores ≥20 on the K10
Exclusion criteria	Clear organic cause for mental health difficulties Alcohol or drug dependence Currently in mental health crisis People outside of England.	Clear organic cause for mental health difficulties Alcohol or drug dependence Currently in mental health crisis People outside of England.
Data	The experiences of young people will be presented through many forms of communication (and modalities of data collection as above), using their preferred vocabularies to communicate their personal stories and experiences	The experiences of young people will be presented through many forms of communication (and modalities of data collection as above), using their preferred vocabularies to communicate their personal stories and experiences.

ADHD, Attention Deficit Hyperactivity Disorder; LGBTQIA+, lesbian, gay, bisexual,transgender, queer/questionning, intersex, asexual and people of identities not explicitly named; NHS, National Health Service.

Three experience-driven research approaches underpin our use of narrative inquiry: (a) increased emphasis on reflection, (b) increased attention to what individuals know, how they think and how they make decisions and (c) letting people tell their stories and share experiences. The experiences of young people will be presented through many forms of communication (and modalities of data collection above), using their preferred vocabularies.

Intersectionality theory: Our approach aligns with an intersectional conceptual framework, which will provide a more comprehensive explanation for the convergence of multiple types of disadvantage.^35^ We will illuminate experiences at intersections of disadvantage that are often overlooked in typical analyses due to assumptions of what is known about young people. In this way, an intersectional approach attempts to be corrective, to inform the design of the intervention and codesign approach so that the content is tailored for those whose voices are traditionally not heard. These groups include young people living in precarity and at the intersections of identity, experience and place. Second, intersectional methods will attempt to illuminate societal systems of disadvantage facing diverse young people and the structural realities that shape their mental health. The implementation of intersectionality approaches (both as a lens and method) pays close attention to existing barriers such as sociocultural and socioeconomic disadvantage and the categorisations of subordination and the persistence of hierarchies^35^ that expose some vulnerable young people to persistent systemic inequality.^36^

By adopting an intersectional framework,^37^ we set aside the use of one-dimensional approaches to understanding inequities that are often adopted in studies exploring diverse young people. Instead, we focus on more dynamic complex analyses that draw attention to how relationships, social hierarchies and structures impact the experiences of young people positioned within their locale or intersect.^38^

### Study participants

WP1: We will select via purposeful sampling up to 100 young people aged 16–24 years who are currently experiencing, or over the last year have experienced a mental health problem from communities, NHS waitlists and online. We will focus recruitment on under-represented young people by ethnicity, gender identity, sexual orientation, neurodiversity (including Attention Deficit Hyperactivity Disorder [ADHD]), geography, occupation, education and deprivation. This sample size of 100 was estimated alongside young people to ensure sufficient sampling of these diverse groups of young people. This will be monitored alongside young people throughout the study.

Recruitment (see table 2) will focus on communities rich in under-represented young people and NHS waitlists in Sheffield, Oxfordshire, Cornwall/Devon, communities in Blackpool and Liverpool and online contact from any region of England. Young people will be invited to complete a questionnaire and then be sampled purposefully based on under-representation, mental health difficulties, use of arts and culture and previous help-seeking.

**Table 2:** Recruitment and sampling details per study

Work package	Number of participants	Sampling strategy	Approach	Region
Work package 1 (WP1)	100	Purposeful	Communities (including NGOs rich in under-represented young people) NHS waitlists Online	NHS and community- Sheffield, Oxfordshire, Liverpool, Cornwall/Devon Community only—Blackpool Online—nationally
Work package 2	30	Purposeful	University of Plymouth’s Cornwall Intellectual Disability Equitable Research (CIDER) centre Those recruited in previous studies, who are eligible and consenting to further contact (but not previously selected)	Regional to the CIDER centre (Cornwall) and as per WP1

NGO, Non-governmental organisation; NHS, National Health Service.

WP2: We will select via purposeful sampling up to 30 autistic young people aged 16–24 years with current or previous mental health difficulties. For WP2, the threshold for inclusion is level 2 autism defined in Diagnostic and Statistical Manual of Mental Disorders 5^39^ as ‘requiring substantial support’, indicating marked deficits in verbal and non-verbal social communication skills, social impairments, limited initiation of social interaction and inflexibility of behaviour or other restricted repetitive behaviours and interests that interfere with functioning. Autistic young people with less significant impairment who are otherwise eligible can be included in WP1. Eligible and consenting autistic young people will be purposefully sampled on the basis of under-representation, by ethnicity, sexual identity and orientation, geography, occupation, education, deprivation, mental health difficulties and previous help-seeking. We will recruit via the University of Plymouth’s Cornwall Intellectual Disability Equitable Research (CIDER) centre, a partnership between the University of Plymouth and Cornwall Partnership NHS Foundation Trust.

### Patient public involvement

There are different ways in which young people from under-represented backgrounds can be involved in mental health research. It is therefore important to differentiate between different roles and how they contribute to the research. Particularly as this research has been designed to be implemented in a collaborative manner, doing the research with the participants rather than to them. These young people will be given the opportunity and support to contribute to outputs including academic papers. Young people have been part of the core research team feeding into decisions on a weekly basis regarding methods, recruitment, sampling and analysis. PPI, peer researchers and youth coordinators will receive training and support through the studies depending on their role, including independent external mentorship for the public coinvestigator. In this project, we have the following roles that are filled by people with relevant lived experience.

- Patient and public involvement coapplicant—LC was involved in developing the research plans, is a coinvestigator and is an integral part of the PPI leadership team.
- Youth co-ordinators will work with LC as a team to organise and co-ordinate the engagement of young people throughout the project and contribute to PPI activities.
- Youth peer researchers will be recruited flexibly through different work studies to contribute to the implementation of the research—for example, interpreting findings in the qualitative work.
- ORIGIN Research Advisory Group (ORAG)—up to 12 young people to consult on how the research is conducted and delivered at key stages ensuring youth-informed approaches to understanding under-representation, agency and ethical considerations, youth-informed engagement and dissemination.
- Youth codesigners—young people contributing to finding and crafting stories for the intervention.

### Participant identification and recruitment

Recruitment will be through communities rich in under-represented young people (focussing on Sheffield, Oxfordshire, Cornwall/Devon, Blackpool and Liverpool) and NHS waiting lists. This will include utilisation of NHS and social care agencies, as well as NGOs, and youth services, and research networks, digital health networks, CAMHS networks, public health agencies and local authorities, creative and cultural industries, as well as online. We will create attractive recruitment posters and materials which we will circulate widely. This may contain animation and/or talking heads from our ORAG and research team, explaining the ethos, research aims and further materials covering more specific aims, objectives, benefits and potential harms, linked to electronic screening and consent procedures and links. This will be done using open adverts, and we can offer guidance and support through specific agencies, where language skills/differences or disabilities may affect participation. Each local venue (Sheffield, Oxfordshire, Cornwall/Devon and Liverpool/Blackpool) will have a local principal investigator (PI). There will also be a safeguarding lead who, with the PIs, will support national non-regional recruitment online through social media. We will liaise with local health and social care and safeguarding systems where necessary.

There are two ways in which young people may be recruited to take part. The first is identification online. We will advertise the study online. We will target online organisations and charities nationally as well as those located in Sheffield, Oxfordshire, Cornwall and Devon, Blackpool and Liverpool with high levels of engagement of under-represented populations and work closely with them to raise awareness about the project and facilitate people to take part who may not traditionally participate in research. We will also use youth-informed methods of reaching under-represented young people online, such as adverts on social media. When potential participants access the study online, they will read the relevant participant information sheet (PIS), be given the opportunity to self-identify initial eligibility (ie, currently experiencing—or over the past year have experienced—a mental health problem (eg, feeling very sad or anxious a lot of the time) and to consent to participation. For WP1, full participation will be subject to purposeful sampling by the research team. Participants will be given the opportunity to be contacted for further studies within and beyond ORIGIN if not selected for this particular part of the study. A member of the research team will then be in touch to confirm ongoing interest for those selected via purposeful sampling and arrange qualitative data collection. For WP2, only those eligible will be approached.

The second is via NHS waitlists. We will work closely with Clinical Research Network teams to sample demographics that are not sufficiently represented from our online recruitment. As these people will be on waitlists, they will not have engaged in an NHS service yet and not have an allocated care team. The PI in each location will enlist relevant staff from the research department in the Trust to send out the advertising materials and links to potential participants alongside contact details for the central research team in case potential participants have questions.

### Screening and eligibility assessment

Details of recruitment and sampling for both WP1 and 2 are outlined in table 2. WP1 will have two steps to ensure purposeful sampling.

Step 1—Initial eligibility and consent—young people who self-identify as being aged between 16 and 24 years old, living in England and who think that they currently have or have had a mental health problem (such as anxiety or depression) over the past year and do not have any of the exclusion criteria (clear organic cause for mental health difficulties, alcohol or drug dependence, currently in mental health crisis) will be given the PIS and the opportunity to give online consent. Once online consent is given, they will have the opportunity to complete an initial questionnaire which includes items on demographics, mental health, help-seeking, use of online arts and culture, a subjective rating scale of socioeconomic status and the Kessler Distress Scale (K10). Text will be carefully crafted alongside young people, making it clear to participants that completing the questionnaire will not guarantee them an interview (but that as stated above, they may consent to being contacted for further studies within and beyond ORIGIN).

The K10 will be used as it strongly discriminates between community cases and non-cases of mental disorders identified by a structured clinical interview.^40^ The K10 comprises 10 questions inquiring about the frequency of depressive and anxiety symptoms with reference to the previous 4 weeks. Each item is rated on a 5-point Likert scale (1=none of the time to 5=all of the time) and scores added to a possible range of 10–50, with higher scores reflecting higher levels of psychological distress. The K10 is one of the most widely used mental health screening instruments^41^ and demonstrates good properties with regard to validity, reliability^41^ and sensitivity to change.^42^ A K10 score of 20 or more is defined as clinically significant distress.

Step 2—Individuals who are eligible and selected via purposeful sampling will be contacted via their preferred contact method and invited to meet with a member of the research team. Consent will be reaffirmed at every interaction. We will coproduce the invitation with our young people, bearing in mind that there is a wide range of barriers to participation for this diverse sample to be considered. This is subject to Research Ethics Committee (REC) approval prior to use.

WP2 seeks to recruit a smaller sample size than WP1. The sample size was chosen in discussion with young people and considered potential challenges in recruiting level 2 autistic young people (ie, those requiring substantial support) and recruitment being restricted by geographical region to ensure the possibility of in-person interviews. Only those who are eligible to participate will be approached. For WP2, we aim to recruit 30 young people with level 2 autism. Recruitment will be conducted via the CIDER centre (part of Cornwall NHS trust).

### Informed consent

Consent will be received online in the first instance when completing the demographic questionnaire that will inform the sampling. Participants will have received the PIS, developed with patient and public involvement (PPI) in accordance with best practice and including clear statements about confidentiality, data access, inclusion criteria and online consent. Online consent procedures will be undertaken by clicking through to the next page after reading the information sheet. An ID number will be allocated at the point of consent that will then be used for all data collection. Participants aged 16–18 years will be considered competent youths. All participants may contact the research team if they need assistance completing the form or would prefer to participate in person. Contact information for the researchers will be provided in the information sheet in case of any further queries. We will test the form and add validation fields so that participants cannot progress if their consent form has not been properly completed.

### Data collection

Youth peer-researchers will be involved in collecting data, interpreting results and will inform the overall analytic strategy, scope and depth. This means tailoring the research process to optimally engage and support them to participate, as well as ensure their perspectives (at multiple intersects of identity, experience and place) are fully heard and accommodated in the analytic, interpretive and dissemination process. We will codesign data collection processes with young people. This will be iterative and responsive to the needs of the participants. The nature of narrative interviews incorporates flexibility that allows for the participant to conduct the interview in multiple sittings using a method that is comfortable for them.

Following an opening statement inviting the participant to tell their story in their own way, interviews will be audio recorded (not video recorded) and transcribed by the research team. Not consenting to being audio recorded will not affect eligibility to take part in the study and we will explore alternatives to document the interviews on a case-by-case basis. Audio recordings will be kept securely until the study end date for data verification purposes. Transcripts will be stored in a deidentified form with all personal identifiable data removed.

### WP1: gathering experiences

After viewing the information sheet and providing informed consent, participants will be asked to complete a questionnaire. From this information, the research team will then sample up to 100 people to gain an information-rich, diverse sample and aim for maximum contrast. Those selected will be contacted by the research team and invited to be interviewed at a time convenient to them. The interview will be a narrative interview allowing participants to share their story at a pace and in a format that is comfortable to them. Working alongside youth peer researchers with lived experience, interviews will be offered face to face, and we will facilitate online interviews where requested.

### WP2: gathering experiences (Autism)

This study will be the same as WP1, however, adjustments will be made to enable people with level 2 autism to participate authentically. This will entail providing materials in alternative formats. All research materials will be ethically approved prior to use. We will ensure that we have representation from people with autism in all levels of the research, including the ORAG (comprising young people aged 16–24 years), to ensure that this study is conducted in a way that enables people to meaningfully participate and share their experiences. We have three coapplicants with lived experience of autism, as well as peer researchers and the ORAG whom we will be working alongside to deliver this study effectively.

### Early discontinuation, study end, safety reporting and adverse events

Participants may withdraw from the study at any time. They may choose to discontinue the study at any time-point without explanation. Any reasons given for withdrawal will be logged. If the participant is withdrawn due to an adverse event, the investigator will arrange for signposting to local and national mental health support resources. In addition, the Investigator may discontinue a participant from the study at any time if the Investigator considers it necessary for any reason including, but not limited to:

- Ineligibility (either arising during the study or retrospectively having been overlooked at screening).
- Unable to be contacted for follow-up interview.
- Significant protocol deviation.
- Clinical decision.

The type of withdrawal and reason for withdrawal will be recorded in the case report form. If the participant is withdrawn due to an adverse event, the Investigator will arrange for telephone calls as per participant preference until the adverse event has stabilised and will report these to the steering committee. The steering committee will meet prior to recruitment, during active recruitment and prior to the study ending. The end of study is the point at which all the study data has been entered and queries resolved.

Given the nature of the interview—an opportunity to share their experiences guided by the participant, following the principles of narrative interviewing—we do not expect any serious adverse events. However, with the ORAG, we will consider the most appropriate approaches to monitoring, reliable reporting and management of adverse events. Given previous experience, we are anticipating that any adverse events will be non-serious and will be addressed by ensuring appropriate support is available to the individual. We have developed a distress protocol and will ensure that all interviewers undertake adequate training prior to conducting any interviews.

A serious adverse event is any untoward medical occurrence that:

- Results in death.
- Is life-threatening.
- Requires inpatient hospitalisation or prolongation of existing hospitalisation.
- Results in persistent or significant disability/incapacity.
- Consists of a congenital anomaly or birth defect.

From experience of conducting the pilot study, we do not anticipate serious adverse events as a result of taking part in this research. All serious adverse events will follow policies and procedures agreed with the study sponsor (University of Oxford).

### Qualitative analysis

We anticipate that data will be transcribed and explored using a combination of thematic analysis and intersectional analysis.^35^ The exact analysis process will be shaped by young people’s preferences as peer-researchers working with the research team. The thematic content of their experiences will feed into and shape the translational aims of the research to locate the delivery of the rest of the research programme within young people’s preferences and experiences. As we anticipate data from a heterogenous population, thematic analysis alone may not be sufficient to ensure that different voices are fully represented. We will involve young people in the coding of data and interpretation of the findings. We will adopt a process of familiarisation, coding and thematic analysis by constant comparison,^43 44^ using the principles of Framework Analysis to document, organise and manage the data in a matrix.^45^ We regard experience data as shining a light on real events and processes that are of value and are not to be dismissed as subjective experiences. The insight of young people will provide several points of reflection and refinement for the research team. The approach adopted will remain flexible in response to the emerging themes that will surface from the participants. The diverse sample and intersectional approach will provide a rich data set of intersectional mechanisms and processes through which mental health and resilience unfold.

### Data management

Data storage, access, curating and archiving will follow General Data Protection Regulation policies for the protection of personal data. Details regarding data management considerations for both studies are outlined in table 3.

**Table 3:** Data management

	Data	Monitoring	Storage	Archiving
Work package 1 (WP1)	Recruitment log Demographics Selection via purposeful sampling Consent forms Audio recordings and Transcripts	Weekly during data collection	Digital, (eg, Qualtrics) for consent, excel for demographics, stored in a password-protected folder on Microsoft Teams, only accessible by the immediate study team	NHS trusts (for recruitment logs) University of Oxford
Work package 2 (WP2)	Recruitment log Demographics Selection via purposeful sampling Consent forms Audio and transcripts	Weekly during data collection	Digital, (eg, Qualtrics) for consent, Excel for demographics, stored in a password-protected folder on Microsoft Teams, only accessible by the immediate study team	NHS trusts (for recruitment logs) University of Oxford
ORIGIN research advisory group (ORAG)	Meeting notes and reflections	Quarterly	Digital	University of Oxford

## Ethics and dissemination

This study (IRAS project ID 340259) has received ethical approval from the HRA and Health and Care Research Wales (REC reference 24/SC/0083). Using existing ethical frameworks and emergent codesigned approaches, we will provide dynamic processes of engagement, support, consent and information tailoring to needs and contexts. Ethical issues will be reviewed by the ORAG and the Programme Steering Committee regularly.

Dissemination activities are integrated through all phases of the research and aim to maximise how information is shared and measure the impact of the research more broadly. We expect to deliver meaningful impact for secondary beneficiaries, for example, for health, arts, education and policy professionals seeking innovative approaches for tackling mental health issues in young people. We will share knowledge with young people, policy-makers, health professionals, carers, teachers, social workers and people who work in arts and culture. We will produce research papers, blogs, newsletters, webinars, videos and podcasts. The outputs will identify touch points and refine the logic model of how online arts and culture might support the mental health of those from under-represented backgrounds.
