# Supplementary material for: Exploring experiences of mental health challenges in under-represented young people (aged 16–24 years) in England: a narrative inquiry protocol
Source: BMJ Open. 2025 Nov 26;15(11):e098223. doi: 10.1136/bmjopen-2024-098223 (PMC12658539; doi:10.1136/bmjopen-2024-098223)
Supplement: Supplementary file 1 [file bmjopen-2024-098223.reviewer_comments.pdf]

PEER REVIEW HISTORY

BMJ Open publishes all reviews undertaken for accepted manuscripts. Reviewers are asked to complete a checklist review form and are provided with free text boxes to elaborate on their assessment. These free text comments are reproduced below.

ARTICLE DETAILS

Title (Provisional)

Exploring experiences of mental health challenges in underrepresented young people (aged 16–24 years) in England: a narrative inquiry protocol

Authors

Sheriff, Rebecca; Arday, Jason; Shankar, Rohit; Mooney, Roisin; Chandler, Louise; Adams, Helen; Nagy, Lili Z; Farrell, Roger; Fancourt, Daisy; Weich, Scott; Henderson, Catherine; Hassan, Shaima; Langley, Joe; Bhui , Kamaldeep

---

VERSION 1 - REVIEW

**Reviewer**	**1**
**Name**	**Dowell, Anthony**
**Affiliation**	**Wellington School of Medicine and Health Sciences, General Practice**
**Date**	**23-Jul-2025**
**COI**	**None**

As the authors correctly state , the great majority of mental health problems begin before the age of 25 , and both in the UK and internationally there are challenges in providing services and support for people in that age group. It is also true that for these young people certain groups are at increased vulnerability , with ethnicity and poverty being prominent drivers.

Exploration of the views of young people about both causation and solutions is thus helpful, and this paper provides an outline of a work programme that will provide useful information, with a focus on the place of arts and culture as part of a strengths based approach.

Introduction

The introduction appropriately outlines basic features of the epidemiology of mental health problems in young people. The inclusion of aspects such as the impact of the COVID pandemic and the inclusion of Autistic Spectrum Disorder is helpful.

• Young people with ADHD are also a group with additional needs who might be included.

The introduction provides useful referencing as to the potential role of arts and culture input as part of support for young people, and of the importance of narrative methodologies.

It also outlines the impressive prior work undertaken with the 'Ways of Being ' project , indicating that arts and culture engagement was received well with the increased focus on diverse human stories".

The Work packages outlined from the prior work appear feasible based on prior work. The focus on Autism Spectrum Disorder is welcome.

Methods

Mixed methods are appropriate for these work packages, and a lot of thought has gone into recruitment and the relationship between the different work packages.

The focus on intersectionality is important, since the 'convergence of multiple types of disadvantage ' is more complex than a simple addition of ethnicity + poverty + spectrum disorder.

• The emphasis on letting young people tell the stories opens up interesting research challenges in terms of co-design and co-production throughout the research process. The section differentiating between PPI, Peer researchers and Participation shows the collaborative approach by the research team. The presence of research roles filled by people with relevant lived experience is a clear marker of a co-design approach: to what extent will the youth coordinators and peer researchers be part of the research outputs and dissemination eg as authors on publications?

The sampling frame would appear to be broad, taking those with mental health difficulties from communities, NHS waitlists and online. Will there be any 'severity' grading in terms of a threshold for what constitutes a mental health problem?

The multiple approaches to recruitment should provide an both the targeted and diverse population required.

A thoughtful and sensitive approach has been taken to data collection, and clinical safety netting.

Overall the research team are to be congratulated on the production of an impressive protocol.

**Reviewer**	**2**
**Name**	**Kealy, Carmen**
**Affiliation**	**University of Galway, HPRC**
**Date**	**30-Jul-2025**

COI **None**

An interesting and important research project related to mental health in underrepresented youth, which will contribute greatly knowledge in the area.

Some comments to consider

The protocol as is reads more like an ethics application and is overall badly structured. As a result it is incoherent and hard to follow, so please rework

Other points

Introduction

First paragraph:

First sentence needs elaboration and as opening statement too weak. Provide reference to the statement that it is priority for young people etc.

Paragraph 2:

Explain how you define culture. Provide better context, e.g. explain immediately the consultation process that the authors have already engaged in. As mentioned previously, the flow of the protocol is difficult to follow for anyone not familiar with your project overall and the various elements (WPs) you are trying to incorporate

Research question section

Needs lead-in sentence to the work packages as unclear how these are relevant

How did you derive sample size? Why 16-24 year olds? Clearer definitions of underrepresented youth needed (spell them out) in inclusion critera

Did young people choose the methodology you will employ? This needs to be explained/stated

You speak about peer researchers and need to elaborate on how they will be trained etc.

References:

This is a very popular and fast evolving research area at present, so be sure to keep up to date with latest developments and publications

---

VERSION 1 - AUTHOR RESPONSE

Reviewer: 1

• Young people with ADHD are also a group with additional needs who might be included.......

Thank you

We agree
The focus of recruitment aims to capture those with neurodiversity including ADHD. This has now been clarified (p9)

to what extent will the youth coordinators and peer researchers be part of the research outputs.......
Thank you
Our public co-applicant (a young person of the target age group) is a co-author on this protocol, and we acknowledge other youth contributions to this protocol. We will give the opportunity to and support youth coordinators and peer researchers on these studies to achieve authorship on the research outputs. This is now clarified on page 9.

Will there be any 'severity' grading in terms of a threshold for what constitutes a mental health problem?.....
Thank you for this
We agree
We have clarified this is Table 1- 'Identifies as having current or previous mental health difficulties in the past year OR scores =>20 on the K10. We have now also included a clear explanation of the rationale of using the k10 and cut off for severity on page 12

The multiple approaches to recruitment should provide an both the targeted and diverse population required.
A thoughtful and sensitive approach has been taken to data collection, and clinical safety netting.
Overall the research team are to be congratulated on the production of an impressive protocol.......
Thank you for your thoughtful and helpful comments

Reviewer: 2
please rework........
Thank you
We agree
This has now been completed throughout

First sentence needs elaboration and as opening statement too weak........
Thank you
We agree
This has now been amended (p4)

Provide reference to the statement that it is priority for young people etc.........
Thank you

This has now been added (p4)

Paragraph 2:
Explain how you define culture........
Thank you
This has now been reorganised with clearer explanation and reference to consultation

Research question section
Needs lead-in sentence to the work packages as unclear how these are relevant........
Thank you
This has now been edited and clarified and terminology amended so it is clear that only WP1 and 2 are covered here.

How did you derive sample size?
Thank you
This has now been edited and clarified (p9)

Clearer definitions of underrepresented youth needed (spell them out) in inclusion critera......
Thank you
This has now included in Table 1 (p8)

Did young people choose the methodology you will employ? This needs to be explained/stated. You speak about peer researchers and need to elaborate on how they will be trained etc......
Thank you
We agree
This has now included in pages 9/10

References:
This is a very popular and fast evolving research area at present, so be sure to keep up to date with latest developments and publications
Thank you
We agree
This has been updated
